# Osteolytic Bone Metastasis: Different Radiotherapy Fractionation Schedules Compared Clinically and Radiographically

**DOI:** 10.3390/curroncol31060233

**Published:** 2024-05-29

**Authors:** Zoi Liakouli, Anna Zygogianni, Ioannis Georgakopoulos, Kyriaki Mystakidou, John Kouvaris, Christos Antypas, Maria Nikoloudi, Vasileios Kouloulias

**Affiliations:** 1 Radiotherapy Unit, 1st Department of Radiology, Medical School, Aretaieion University Hospital, National and Kapodistrian University of Athens, 11528 Athens, Greece; azygogianni@med.uoa.gr (A.Z.); ioangeo@med.uoa.gr (I.G.); kmystaki@med.uoa.gr (K.M.); ioankouvaris@med.uoa.gr (J.K.); cantypas@med.uoa.gr (C.A.); mnikoloud@med.uoa.gr (M.N.); 2 Department of Clinical Radiation Oncology, Medical School, AΤΤΙΚOΝ University Hospital, National and Kapodistrian University of Athens, 12462 Athens, Greece; vkouloul@med.uoa.gr

**Keywords:** osteolytic bone metastasis, radiotherapy, quality of life, bone density, remineralization

## Abstract

The purpose of this study is to compare three commonly used radiotherapy fractionation schedules for bone metastasis in terms of clinical and radiological effectiveness. A total of 93 patients with osteolytic bone metastasis were randomized to receive 8 Gyin a single fraction (group A), 20 Gy in 5 fractions (group B) and 30 Gy in 10 fractions (group C). Changes in bone density were measured using the Relative Electron Density (RED) type corrected by Thomas (pe = HU/1.950 + 1.0), where HU is Hounsfield Units. Pain response was assessed according to the Brief Pain Inventory tool. Quality of life was estimated using the EORTC QLQ-C30 and the MD Anderson Symptom (MDAS) tools.After RT, RED, together with the parameters of EORTC QLQ-C30, MDAS and SAT, significantly increased in all groups (*p* < 0.001).Specifically, the increase of RED was higher in group C compared to group Athree months post-RT (*p* = 0.014). Group C was also superior to group A in terms of QoL and BPI three months post-treatment. Multifractionated radiotherapy for osteolytic bone metastasis is superior to single fraction radiotherapy in terms of improvement in quality of life and bone remineralization three months post-RT.

## 1. Introduction

Different cancer types metastasize to specific organs, and this has led many investigators to search for a mechanism responsible for this preference. Paget, in 1989, postulated the theory of “seed and soil” in order to explain the increased incidence of liver, lung and bone metastasis caused by breast cancer. In accordance withthe observations from other researchers, he concluded that the primary tumor, which is the “seed”, most commonly metastasizes to tissues that are the appropriate “soil” [1]. A total of 65 to 75% of patients with advanced metastatic prostate and breast cancer present with bone disease. Similarly, 60% of patients with thyroid cancer, 40% with lung and bladder cancer and 25% of patients with renal cell carcinoma have bone metastasis [2]. Patients with bone metastasis suffer from pain, pathologic fractures, hypercalcemia and spinal cord compression, especially those with osteolytic type of bone disease [3,4]. Radiotherapy is very commonly used for the palliation of pain; multiple trials have shown that different fractionation schedules, from single-fraction to multi-fraction radiotherapy, are comparable in terms of pain palliation and the duration of this palliative effect [5,6,7,8,9,10,11,12,13]. More importantly, radiotherapy has a positive impact on the global quality of life ofthese patients [14]. Apart from clinical response, it also leads to remineralization of the affected bone, which is apparent in computed tomography images after a period of time of approximately 2 months from completion of treatment [15]. Changes in bone density and the effect toquality of life have been examined in trials. The tools that have been used for quality-of-life metrics were not uniform, and a comparison of both bone density and quality of life has not been made. A comparison of the most commonly used fractionation schedules regarding both quality of life and changes inbone density could identify a more effective schedule that could bring a change in everyday clinical practice.

## 2. Materials and Methods

### 2.1. Ethical Statement

In terms of ethical approval, all procedures performed in studies involving human participants were in accordance with the ethical standards of the institutional and/or national research committee and with the 1964 Helsinki Declaration and its later amendments or comparable ethical standards. The study was approved by the local ethics committee.

### 2.2. Patients

The inclusion criteria for the study were (1) age > 18 years old, (2) biopsy-proven cancer, (3) osteolytic bone metastasis, (4) ECOG Performance Status 0–2. There could be no change in the systematic treatment of the patients 4 weeks before and 4 weeks after radiotherapy to avoid interruption with the results of radiotherapy, according to other randomized trials for bone metastasis.

**Figure 1:**
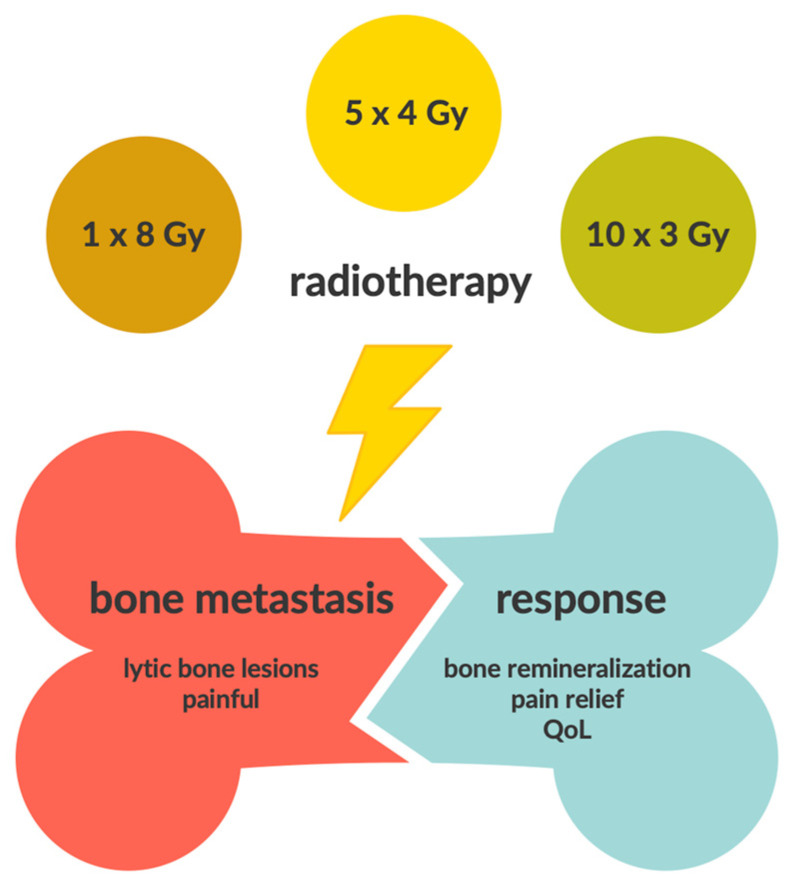
Trial schema.

The trial schema is shown in Figure 1. Patients were randomly assigned to one of the following groups: 8 Gy in one fraction (group A), 20 Gy in five fractions (group B) and 30 Gy in ten fractions (group C). Under this scope, 28 patients in group A, 33 patients in group Band 32 patients in group C were included in the analysis. The median age was 70 years in group A, 60 years in group B and 68 years in group C. The majority of the patients with breast cancer were ER/PR positive, as expected. The characteristics of systematic treatment (chemotherapy, hormonal therapy, bone modifying agents) were similar in all three groups. The patients’ characteristics are shown in Table 1.

### 2.3. Radiotherapy

All patients underwent a computed tomography with 5mm slice thickness for treatment planning purposes. Data were transferred to the treatment planning system (Oncentra), while a 3D conformal technique was used for the irradiation. Gross Tumor Volume (GTV) was the osteolytic lesion contoured in bone window, and Clinical Target Volume (CTV) was the affected bone in case of vertebral metastasis and partial bone in case of other bone types (e.g., femur, iliac), according to the ESTRO ACROP guidelines for external beam radiotherapy of patients with bone metastasis [16]. A Planning Target Volume (PTV) margin of 5mm was applied to take into account uncertainties in daily setup. The dose was calculated at the isocenter according to International Commission on Radiation Units and Measurements (ICRU point). The dose within the PTV ranged between 95% and 107% of the isocentric dose, according to ICRU recommendations.Radiation treatment was delivered using a 6 MV Oncor lmpression linear accelerator(Siemens, Germany)equipped with an 82 multileaf collimator (leaf thickness, 1 cm). For quality assurance purposes, double-exposure portal films were obtained weekly and compared with the corresponding digitally reconstructed radiograph from initial simulation. 

**Figure 2:**
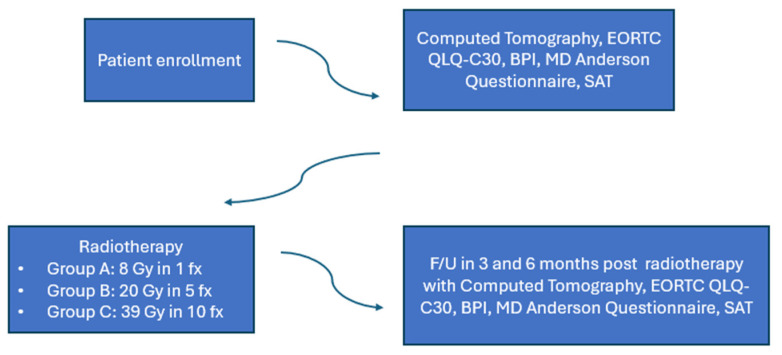
Trial design.

Patients were evaluated at 3- and 6-months post radiotherapy. At each follow-up visit, they underwent a physical examination. A computed tomography scan of the treated bone metastasis was obtained for evaluation and calculation of remineralization of the bone. Pain response was assessed with the Brief Pain Inventory tool. Quality of life was estimated using the EORTC QLQ-C30 ver. 3.0and the MD Anderson Symptom tools, greek validated. Self-Assessment Treatment tool was used to estimate the patients’ satisfaction withradiotherapy treatment. The trial design is shown in Figure 2.

### 2.4. Data and Statistical Analysis

Changes in bone density were calculated using the Relative Electron Density (RED) type corrected by Thomas (pe = HU/1.950 + 1.0), where HU is Hounsfield Units. For pain response, a Brief Pain Inventory questionnaire was used, a tool that estimates pain level during the past 24 h and its effect on daily activities through 11 questions. EORTC QLQ-C30 questionnaire is designed to measure cancer patients’ physical, psychological and social functions. It consists of 9 sections, 5 relating to functionality (physical, emotional, social, mental functions and activities), 3 relating to symptoms (pain, fatigue and nausea/vomiting) and 1 for global quality of life and health. MD Anderson Symptom Questionnaire evaluates patients’ symptoms during the past 24 h and their interference with daily living.

The whole analysis was performed by using the SPSS 8.0 package (SPSS, Inc., Chicago, IL, USA). Non-parametric Kruskal–Wallis test was used for statistical analysis, while a *p* value of less than 0.05 was considered significant.

## 3. Results

**Figure 3:**
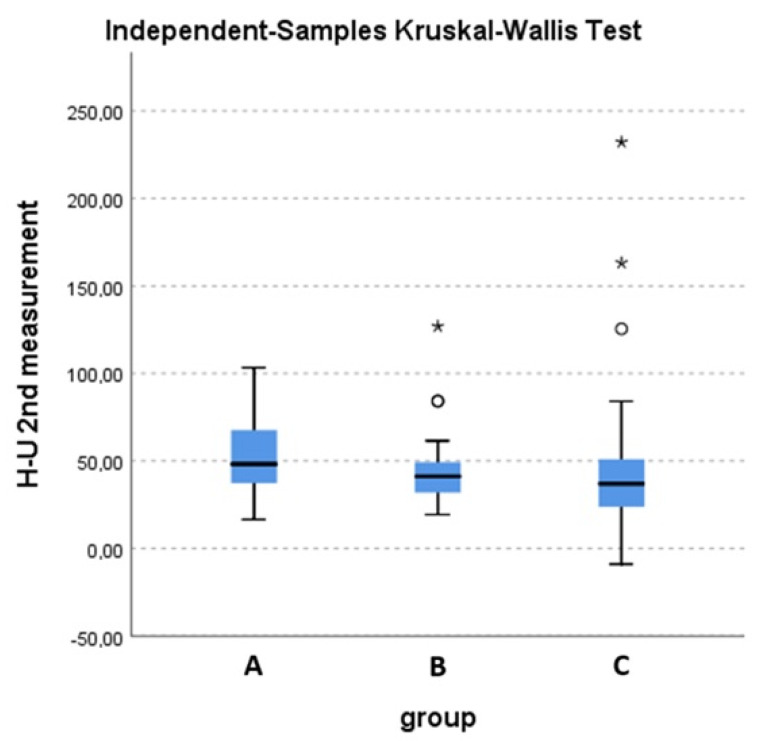
Overall difference in HU units in the second RED measurement between the three groups. Kruskal-Wallis test (*p* = 0.047). A statistically significant difference is observed between groups A and C (*p* = 0.014). * and o stands for outliers.

Ofthe 93 patients, 13 had disease progression after the first three months and were lost in follow-up (2 in group A, 5 in group B and 6 in group C). In group C, one of the patients suffered a fracture of the irradiated bone. After RT, RED, together with the parameters of EORTC QLQ-C30, MDAS and SAT, significantly increased in all groups (*p* < 0.001) at three and six months. When compared, the increase inRED was higher in group C compared to group A three months post-RT, which was statistically significant (*p* = 0.014), as shown in Figure 3. Group C was also superior to group A in terms of QoL and BPI three months post-treatment. In greater detail, group C was superior in terms of pain reduction, the interference of pain with general activity and quality of life, as well as the interference of physical condition with social activity. No significant differences were noted between groups B and C in terms of RED and QoL parameters. Finally, none of the patients in each group experienced a worsening of the ECOG Performance Status during treatment. Specific results of the questionnaires are shown in Table 2 and Table 3, by group, for baseline and 3 and 6 months post-RT. 

## 4. Discussion

Bone metastasis affects a great number of cancer patients worldwide. According to the Health Care System in the USA (Ministry of Health, Washington, DC, USA), on 31 December 2008, 280,000 patients with cancer had bone disease. The management of bone disease requires the collaboration of a multidisciplinary team in order to relieve the pain, improve quality of life and reduce skeletal morbidity. It is well established that radiotherapy is one of the most effective treatments for palliation of pain [17], positively affecting the quality of life [18] and is cost-effective [19].

Apart from clinical improvement, radiotherapy also has an impact on the bone density of affected bones, which is of significant importance, especially in the case of osteolytic metastasis, which is often complicated with pathological fractures. The effect of radiotherapy on bone density was shown in a study by Kouloulias et al. in patients who received disodum pamidronate in conjunction with radiotherapy. Bone density changes were quantitatively measured in plain radiographs with the use of gray-level histograms. In osteolytic metastasis, the mean gray value is decreased, and the mean energy is increased. After radiotherapy, an increase in mean gray value was observed and a decrease in mean energy, indicative of response to treatment [20,21]. There were several disadvantages tothe use of plain radiographs, especially in areas with organ movement, such as the abdomen and thorax [22]. Computed tomography, on the other hand, is an easy and fast examination that is most commonly used for staging and monitoring cancer patients. It has the ability of the multi-planar reconstruction of images in a specific window for bone. Vasiliou et al. measured changes in bone density in computed tomography images of patients receiving ibandronate in conjunction with radiotherapy and reported an increase in bone density by 73.2% in 10 months, measured in Hounsfield Units [23,24].

Several studies have examined the potential difference in bone density changes and pain relief between radiotherapy schedules. In a study by El-Shenshawy et al., 150 patients were randomized to receive 8 Gy single fraction, 20 Gy in 5 fractions and 30 Gy in 10 fractions. Patients were evaluated three months post-radiotherapy. Results from this study showed that there is no difference in pain relief, with 78% of patients in the single fraction group reporting some degree of pain relief and 79% of the patients in the multi-fraction groups, respectively. Bone density changes, though, were statistically significantly different between the three groups. The median percentage (%) change in bone density in the 8 Gy group was 105, 128 in the 20 Gy group and 181 in the 30 Gy group. The change in bone density was correlated with pain relief. Patients reporting complete pain relief had a median % change in bone density of 243;in those with partial pain relief, the change was 178, and in patients that reported an increase in the level of pain, the change in bone density was 79 [25].

In another study by Koswig and Budach, patients received either 8 Gy single fraction or 30 Gy in 10 fractions. Pain relief was similar between the two groups, but bone density changes were statistically significantly different. Bone density increased by 173% in the 30 Gy group and by 120% in the single fraction group [26]. 

There was an improvement in quality of life, although that was not well studied, and the tools that were used were quite variable. In a study by Steenland et al., 1171 patients with bone metastasis were randomized to receive 8 Gy in one fraction versus 24 Gy in six fractions. Quality of life was estimated with the Rotterdam Symptom Check List tool, which consists of 38 questions covering 3 sections:physical, psycho-social and daily activities. The analysis of the repeated measurements showed that there was no difference in the improvement in generalquality of life between the two schedules [27].

Hartsell et al., in a randomized phase III trial, compared 8 Gy in one fraction versus 30 Gy in ten fractions [28]. The tool used for the estimation of quality of life was the Health Utilities Index III, which measures eight parameters, namely vision, hearing, speech, mobility, emotion, mental functions, pain and skills, on a five- to six-point scale. Ofthe 893 patients that were included in the trial, complete data on quality of life were obtained from363 of them. Generalquality of life improved significantly in both groups, and there was no difference between the two groups. 

In another randomized study by Kaasa et al, 8 Gywascompared with 30 Gy. Pain relief was the primary endpoint and was estimated with a five-point scale, while quality of life was a secondary outcome [29]. EORTC QLQ-C30 questionnaire was used in this study. Improvement in quality of life was similar between the two groups.

Interestingly, in a very recent trial from Maemoto et al., the deterioration of ECOG PS during radiotherapy treatment was directly correlated with survival, suggesting that, in these cases, the discontinuation of treatment should be considered [30]. None of the patients in our study showed signs of symptoms worsening; on the contrary, a significant proportion experienced improvement in pain symptoms even before the completion of treatment.

None of the above-mentioned studies has compared both bone remineralization and quality of life. Furthermore, the tools used for quality-of-life estimation were variable. In our study, a comparison with both clinical and radiological criteria was performed. The EORTC QLQ-C30 questionnaire was used for quality of life estimation, which is a well-validated and widely used tool. 

EGFR status for lung cancer patients was not recorded in our study, although recent studies indicate improved treatment response for patients with EGFR mutation receiving TKIs [31]. Changes in systematic treatment, though, were not allowed 4 weeks before and 4 weeks after radiotherapy to avoid the interruption of systematic treatment with the results. The majority of the patients with breast cancer were ER/PR positive, as expected since this population has a higher incidence of bone metastasis. Systematic treatment was similar in all three groups in terms of chemotherapy, hormone therapy and bone-modifying agents. The statistically significant results three months post-RT are in accordance with the results from other studies that show that the maximum palliative result of radiotherapy is noticed twelve weeks post-treatment [29]. The non-statistically significant difference six months post-treatment could be due to possible changes in systematic treatment, the loss of patients during follow up and the fact that maximum remineralization is observed three to four months post-radiotherapy. 

According to our findings, it is shown that ten-fraction radiotherapy compared to single fractions is superior in parameters associated with bone density, the interference of pain with daily activities and generalquality of life. Patients were equally satisfied with the different fractionation schedules while attempting radiotherapy ten consecutive times didnot affect them negatively.However, our study has some limitations. Some patients were lost to follow up, due to disease progression, possibly affecting the evaluation at six months. In any case, most trials on bone metastasis evaluate patients at three months. Its’ significance on the other hand, stands in the objective criteria used for treatment response and the comparison with both clinical and radiological criteria. Thus, more prospective trials stand in need in order to extract safe results.

## 5. Conclusions

As is shown in our study, ten-fraction is superior to single-fraction radiotherapy in terms of bone remineralization, a result consistent with previous studies. It is also shown that improvement in bone density is correlated with improvement in the quality of life of patients, a parameter that has not been uniformly studied before. Multiple fraction radiotherapy may be the preferable option for select patients with painful osteolytic bone metastasis, especially those with good performance status and better prognosis.

## Figures and Tables

**Table 1:** Patients’ characteristics.

Patient Characteristics	Group A (28)	Group B (33)	Group C (32)
Sex			
Male	15	16	16
Female	13	17	16
Age, years range (median)	40–84 (70)	47–81 (60)	40–84 (68)
Primary			
Lung	10	18	7
Breast	14	9	16
ER/PR+	10	7	13
CerbB2−	12	6	13
Other histologies	4	6	9
ECOG PS			
0	9	12	10
1	10	12	12
2	9	8	10
Hormone therapy	9	6	10
Chemotherapy	15	18	19
Bone modifying agent	15	11	19

**Table 2:** Mean values of RED, along with overall values of QLQC-30, BPI, MDAS and SAT questionnaires by group of patients at baseline and 3 and 6 months post-RT.

	Baseline			3 MonthsPost-RT			6 Months Post-RT		
Group	A	B	C	A	B	C	A	B	C
RED	66.5	67.8	66.7	113.9	115.6	114.5	190.7	192.0	191.2
QLQ-C30	4.6	4.4	4.5	5.3	5.4	5.4	5.6	5.7	5.7
BPI	4.72	4.64	4.66	3.01	2.97	2.93	2.24	2.03	2.13
MDAS	1.48	1.50	1.47	1.91	0.86	0.89	0.59	0.56	0.57
SAT	3.83	3.91	3.84	4.09	4.08	4.13	4.26	4.27	4.32

**Table 3:** QLQC-30 questions 9, 27 and BPI questions 9A, 9G by group of patients at baseline and 3 and 6 months post-RT.

	Baseline			3 Months Post-RT			6 Months Post-RT		
Group	A	B	C	A	B	C	A	B	C
QLQ-C30, question 9	2.68	2.67	2.66	2.14	2.15	2.14	2.06	2.08	2.07
QLQ-C30, question 27	1.38	1.35	1.37	1.15	1.16	1.44	1.03	1.04	1.03
BPI, question 9A	5.44	5.43	5.38	2.97	2.86	2.97	2.09	2.08	2.06
BPI, question 9G	5.36	5.35	5.27	2.53	2.48	2.52	2.11	2.10	2.08

Abbreviations. QLQ-C30 question 9: “During the past week have you had pain?”; QLQ-C30 question 27: “during the past week has your physical condition interfered with your social activities?”; BPI, question 9A: “how, during the past 24 h, has pain interfed with your general activity?”; BPI, question 9G: “how, the past 24 h, has pain interfered with your enjoynment of life?”.

## Data Availability

Data for this trial can be found in the archives of Aretaieio University Hospital, National and Kapodistrian University of Athens.
